# Reconsidering Planning and Management of Medical Devices Procurement in Public Health Services in Cyprus

**DOI:** 10.5539/gjhs.v7n6p205

**Published:** 2015-04-16

**Authors:** Mamas Theodorou, Marina Georgiou, Athanasios Nikolentzos, Thalia Bellali

**Affiliations:** 1 Open University of Cyprus, 33 Yiannou Kranidioti, 2220, Latsia, Cyprus; 2 Ministry of Health, Pharmaceuticals Services, 15 Polifimou, 2033, Nicosia, Cyprus; 3 Alexandreio Technological Educational Institute of Thessaloniki P.O BOX 141, GR-57400, Thessaloniki, Greece

**Keywords:** medical devices, hospital procurement, Delphi method, Cyprus

## Abstract

Hospital procurement is a crucial field for any health care system, not only for economic reasons but also for reasons related to the quality and safety of the services provided. That is why the process of procurement is, in most countries, governed by a strict legal framework and policy mechanisms. This study investigates the problems and inefficiencies associated with the procurement of medical devices in public hospitals in Cyprus and formulates empirically documented proposals for improvement. Using the Delphi method, a group of 38 experts approach the procurement system in Cyprus from different angles, achieving high rates of consensus on 35 different statements on the weaknesses and problems of the current medical device procurement system, as well as presenting proposals and recommendations for improvement. The findings are highly valuable for future policy initiatives in Cyprus in the light of the economic crisis and the expected implementation of the new General Health Insurance System (GeSY), which the Government of the Republic of Cyprus and the Troika has agreed.

## 1. Introduction

The use of medical technology produces remarkable results for health-related problems and the improvement of patients’ quality of life, while is associated with cost reductions. Nevertheless, its expeditious introduction and dissemination in health systems were among the key factors associated with the huge growth of total health expenditure in recent years (Mossialos and Le Grand, 1999; Fuchs, 2011). The high cost of acquisition, use and maintenance, the short “life expectancy”, the excessive and often indiscriminate use and the fertile environment for provider-induced demand constitute some of the different aspects of the issue at stake. For these reasons, it is necessary to create an effective evaluation and control mechanism for the procurement of medical equipment, in addition to specific methods for evaluating and controlling the diffusion, the use and the management of such medical technology.

The term “medical technology” encompasses the machinery and equipment, medical devices, drugs and procedures used in the provision of health services, as well as the required supporting mechanisms (Office of Technology Assessment, 1978; Johansen, 1988). The “medical devices” subcategory of medical technology includes a wide range of products, such as machines and devices, implantable material and reagents for in vitro diagnosis, as well as consumable materials and disposable products, such as dialysis filters.

The procurement procedures for medical devices differ from country to country. In England, procurement is carried out by the NHS Trusts and the Primary Care Trusts, with the technical assistance and support of specialized units of the Department of Health (Boyle, 2011). In systems that are more liberal, medical procurement is carried out through the formation of large hospital purchasing alliances with the aim of increasing their purchasing and negotiating power, thereby achieving lower prices and economies of scale (Burns & Lee, 2008). The procurement procedures for pharmaceuticals and medical devices in Greece have recently changed with the establishment of the Health Procurement Committee, which is responsible for receiving requests, organising calls for tender and creating a Price List Observatory for price comparisons, greater transparency and cost control (Dervenis et al., 2012; Kastanioti et al., 2013).

The hospital procurement procedures in Cyprus rest centrally with two distinctive sections/departments of the Ministry of Health (MoH): the Pharmaceutical Services Department and the Purchasing and Supply Directorate. The planning and decision making draw on a specific legislative framework and the procedures vary (open competitions, call for tenders, simplified procedures, negotiation procedures, etc.). It seems that the two different departments cause unnecessary bureaucracy and create conditions of opacity and delays in the planning and timely meeting of the needs, resulting in an increasingly high level of spending for the procurement of medical technology (and pharmaceuticals), especially when simplified procedures exist.

The objective of this study was to investigate the problems involved in the procurement of medical devices in public hospitals in Cyprus and to elaborate on specific prospects that may soon be realized. In addition, the aim of this study was to formulate empirically documented proposals for improving the procedures in the procurement chain and eventually reducing the costs.

## 2. Method

To achieve the objective of this study, a number of specialists/experts were involved, under the precondition that they have traditionally played an important role in shaping decision-making and have responsibilities in the wider area of procurement (Swayne et al., 2011).

The primary data collection was carried out using the Delphi method, in two rounds, to obtain a selection of views from experts. The Delphi method is a structured research technique of communication, increasingly used for a variety of different subjects, which mainly takes advantage of the views and opinions of experts in a specific field or topic (Twycross, 2001). It is a practical and flexible approach to the development, evaluation and synthesis of views and thereby greatly aids decision-making (Gibson, 1998; Swayne et al., 2011). The Delphi technique is conducted in consequent rounds, two or more, using structured questionnaires, and it is designed to transform the views of a group of experts into reliable consensus statements (McKenna, 1994; Lynn et al., 1998).

In this study, the choice of the Delphi method was considered to be the most appropriate to elicit the views of experts about the effectiveness and responsiveness of the existing procurement system for medical devices, since the main research questions could be better approached by analysing subjective judgements made on a collective basis (Linstone & Turoff, 1975). The method of focus groups, which could alternatively be used, eventually rejected because it creates conditions that favor phenomena of social pressure, individual domination, halo effect, and does not ensure confidentiality. Additionally, such group dynamics raise ethical issues and may limit the usefulness of the data collected.

### 2.1 Panel of Experts

The sample of experts was assembled from a rather heterogeneous group of people who occupy various posts and have different expertise, yet who have as a common characteristic their active involvement in all the different phases and procedures of the procurement chain of medical devices in Cyprus. It is known that a key element of the Delphi method is the heterogeneity of individuals, who may represent diverse backgrounds with respect to experience and expertise. This heterogeneity of the participants must be preserved to assure validity of the results. Based on this approach, the panel included medical doctors and nurses, administrative personnel, MoH executives and suppliers of medical devices. The literature presents several examples showing that the greater the heterogeneity of the panel, the richer the evaluations from different angles of the same topic and the greater the value and quality of the opinions collected (Grobich, 1999).

Forty-five experts were initially selected, officially informed and invited by email to participate in this study. Thirty-eight accepted (a response rate of 84.4%) and formed the panel of experts for this study. The sample of persons was chosen using the expert sampling method, which is a subcategory of purposive sampling that is mostly used in the Delphi technique. The experts were invited to answer questions in two rounds. Table 1 presents the socio-demographic characteristics of the members of the panel of experts.

**Table 1:** Socio-demographic characteristics of the group of experts

Sex	N	%		
Male	26	68.4		
Female	12	31.6		

Profession	N	%		

Doctors	10	26.2		
Nurses	8	21.1		
Executives of the MoH	6	15.8		
Procurement sector officers- MoH	8	21.1		
Suppliers	6	15.8		

	min	max	mean	s.d.

Age	33	80	49.3	11.48
Years of experience	3	50	13.3	9.71

### 2.2 Data Collection

The first step in the process of data collection was to send the Round A questionnaire to the thirty-eight members of the experts’ panel. The questionnaire included five open-ended questions, which the participants answered according to their views on the topic under study. The subject matter of the queries and the actual formation of the questions were decided after investigating the field of procurement of medical devices in Cyprus. To establish the face validity of the questionnaire, a pilot test was conducted amongst eight experts, who subsequently were excluded from the final sample. The feedback from the experts helped in shaping the final content of the questions and their phrasing. Table 2 presents all questions.

**Table 2:** Round A Questionnaire

Questions of round A
1. There is an increasing trend in implementing “simplified procurement procedures” since 2007 and until 2010 (in terms of how often they have been used and the total amount of money spent). How do you justify this trend?
2. What is the main source of “information/influence” for embracing of new materials and new curative techniques, which demand the procurement of specialized products?
3. According to your point of view, which are the three main problems of the existing procurement chain process of medical devices?
4. Which are the strengths (positive) of the existing procurement procedures?
5. Make at least 3 proposals for improving the operation of the procurement chain procedure of medical devices.

The experts returned by email the completed questionnaires within a set period of thirty days. After the collection, a preliminary analysis of the respondents’ replies followed, which served as a base for the formation of the Round B questionnaire. The preliminary analysis led to the formulation of the questionnaire, which consisted of fifty different statements, classified in six topics. This new questionnaire was sent to the participants, who were asked to express their degree of agreement with each of the statements on a five-point Likert scale. The participants had to choose an option from a scale of 1 to 5, where 1 = strongly disagree and 5 = strongly agree.

### 2.3 Data Analysis

The data were statistically analysed with the use of SPSS v. 16. The acceptable consensus benchmark for each statement was set to 75% and above (Keeney et al., 2011).

## 3. Results

### 3.1 Round A

An in-depth descriptive analysis of the replies received from the Round A questionnaire provided the researcher with 464 answers/statements, a certain majority of which recurred amongst many of the experts.

Regarding the first question about the increasing trend of implementing simplified procurement procedures, 110 statements were recorded. Of the experts, 18 indicated that the increasing trend is due to *the lengthy and bureaucratic procedures and the lack of ability to handle open competitions within set timetables*, while 16 of the experts spotted *deficiencies and weaknesses* in the relevant units of the MoH, such as inadequate staffing and delayed detection of needs, because of the absence of information systems at all the levels of the procurement process chain.

*… inability […] to comply with time schedule deliveries* (physician, 37 years old) and *… cancellation of tenders due to the complexity of specifications …* (administrative officer, 34 years old);

*… lack of computerized monitoring system of the stock* (nurse, 55 years) and *… delay in tenders due to reduced personnel …* (administrative officer, 56 years old).

In the second query on the main source of “information/influence” for new materials and techniques that require the procurement of specialized products, 47 statements were gathered. Of the 32 participants, 24 indicated as the main source the >*manufacturing companies* through their representatives in Cyprus, 20 the *medical conferences* and 4 the *Internet* and *training* centres abroad.

For the third question concerning the three allegedly important problems in the existing procurement chain process of medical devices, 121 statements were recorded, and 20 participants indicated as problems *the fact that two units/departments are responsible for the tenders, the lack of staffing and the lack of technical knowledge specifications of the products*. Furthermore, 18 participants cited the *lack of coordination and communication between hospitals and the MoH*.

*… lack of qualified personnel*, *especially in the preparation of specifications* (administrative officer, 37 years old);

*… different units responsible for tenders with reduced staff* (supplier, 39 years old);

*… the fragmented procurement system* (physician, 63 years old);

*…* *lack of communication between hospitals and the Purchasing and Supply Directorate* (nurse, 53 years old).

In the fourth question about the strengths of the existing procurement system, the experts conveyed 56 statements. Among them, 15 participants favoured the *experience, the responsibility and the diligence of the staff working for the procurement units* and *the legislative framework, which ensures transparency and equal treatment of suppliers and promotes healthy competition*.

*The existence of officials […] with experience, responsibility and diligence …* (administrative officer, 62 years old);

*Nothing, apart from the willingness and diligence of some officials…* (physician, 57 years old);

*… the legal framework …* (supplier, 34 years old).

In the last question, the experts were invited to suggest at least 3 proposals for the improvement of the procurement procedures of medical devices. The responses here included 130 statements, 27 of which suggested *better cooperation and coordination amongst stakeholders and authorities*.

*… better cooperation between officials and departments involved* (administrative officer, 56 years old).

*There must be a system of feed-back from all users of the products to the Tender Committee* (supplier, 56 years old).

They also suggested *the establishment of a Committee for the Evaluation/Monitoring of Requests from each Hospital*:

*… setting up a special committee with a coordinating role and the responsibility to assess and filter all requests from hospitals* (administrative officer, 53 years old);

… *any suggested change in technology should be proposed by a competent committee/body* (physician, 40 years old).

Finally, 22 experts suggested creating a *Central Single Unified Procurement Agency* for medical devices under the MoH, and 10 of the experts added the need for *information systems and a coding system* applied to medical devices.

*Creation of a single unified procurement agency at the MoH, staffed with the necessary and qualified personnel* (administrative officer, 60 years).

### 3.2 Round B

The Round B questionnaires and the replies of the experts provided a bulk of 50 different statements, which were assessed by the experts on a scale from 1 to 5. In the end, a consensus rate of over 75% was accomplished for 35 of the statements. This percentage is considered rather high if one takes into consideration the various backgrounds of the experts, their different experience, posts and responsibilities in the procurement procedures and in some cases their fairly conflicting interests. Tables 3 to 7 present the 35 statements and their percentage of consensus.

**Table 3:** Question A-Round B Questionnaire

Q A: What does good practice in the procurement procedures mean for you?	Percentage Consensus
1. Timely diagnosis/detection of needs	100.0%
2. Better planning/management	100.0%
3. Containment of products not used (reduce waste)	100.0%
4. Consistency in-between different stages of the process: communication and update of information at all stages	97.4 %
5. Reducing deficiencies	94.7%
6. Transparency and equal treatment of suppliers	94.7%
7. Ensuring quality products are available in time and at the lowest price	86.8%

**Table 4:** Question B-Round B Questionnaire

Q B: What are the most important problems of the existing procurement process of medical devices?	Percentage Consensus
1. Lack of coordination/communication and setting of priorities both at the level of the various hospital departments, and the relevant departments of the MoH	92.1%
2. Lack of automated stock control system	92.1%
3. Absence of a mechanism for the evaluation of the use and performance of the medical devices purchased	89.5%
4. Lack of information systems at all levels of the procurement and management of medical products	84.2%
5. The existing supply system is time-consuming in terms of bureaucracy and tendering procedures	81.6 %
6. Difficulties in forecasting needs and in timely planning	81.6 %
7. Difficulty to exclude equipment that proved to be problematic, from future competitions	78.9%
8. Existence of two different tender departments at the MoH, which are poorly staffed and without qualified personnel	76.3%

**Table 5:** Question C, Round B Questionnaire

Q C1: Where do you think the increasing trend of implementing simplified procurement procedures is attributed in recent years?	Percentage Consensus
1. In the rather delayed identification of needs	89.5%
2. In the absence of effective management of consumables	86.8%
3. In the lack of trained personnel and the absence of proper management of consumables	86.8%
4. In the poor planning of needs	84.2%
5. In the absence of immediate effective monitoring and documentation of requests	81. 6%
6. In the lack of prioritizing of needs	79.0%
7. In the lack of coordination between the departments of hospitals and relevant departments of the MoH	79.0%
8. The time schedules for open tenders do not allow adequate response to emergency situations and the purchase of small quantities of specialized products	76.3%

Q C2: What is the main source of “information/influence” for new materials and techniques, which require the procurement of specialized medical products?	Percentage Consensus

1. The manufacturers and their representatives in Cyprus	92.1%
2. Medical conferences and/or further/specialized training in foreign centres	86.8%

**Table 6:** Question D, Round B Questionnaire

Q D: What are the strengths of the current system of the supply chain?	Percentage Consensus
1. The application of an information system and the organisation of the tender section at the Pharmaceutical Services Directorate	86.8%
2. The experience, responsibility, and diligence of certain officials at the Supply and Purchase Directorate, who mainly belong to the low levels of hierarchy	84.2%
3. The legislative framework which ensures transparency, equal treatment of suppliers and promotes fair competition	76.3%

**Table 7:** Question E, Round B Questionnaire

Q E: What solutions would you propose for improving the procurement system?	Percentage Consensus
1. Application of information systems at all levels of the procurement process	97.4%
2. Better cooperation and coordination amongst hospitals, and between the responsible sections involved in the procurement of medical devices	97.4%
3. Establishing an Evaluation and Monitoring Committee to deal with the hospitals’ requests in order to achieve timely forwarding and proper planning of needs	97.4%
4. Encoding, enlisting and categorising medical devices	94.7%
5. Auditing procedures for the efficient management of medical devices	94.7%
6. Establishing a Central Unified Body for the procurement of medical devices at the MoH	92.1%
7. Setting up of a Central Selection and Approval Committee for medical devices at the MoH	76.3%

## 4. Discussion and Conclusions

This paper analyses the current situation of the procurement system of medical devices in Cypriot public hospitals and makes suggestions for the improvement of procedures throughout the entire procurement chain. All the suggestions and proposals came from a panel of experts, in the form of consensus statements, using the Delphi method. The aforementioned analysis contributed to mapping the field of procurement in Cyprus and to describing its basic components.

Round A provided 464 statements, which were later summarized in 50 and finally reduced to 35 after the experts’ consensus. These 35 statements include ideas, opinions and suggestions on best practices, the advantages and weaknesses of the current system and proposals to overcome the problems and generally to improve the procurement procedures.

For the experts, best practice in procurement procedures seemed to have various meanings and raised several issues, such as *early diagnosis and the identification of needs, better planning and management to reduce wastage, consistency in various stages of the procurement procedure, transparency issues and equal treatment of suppliers, as well as ensuring quality products at low prices*. The aforementioned characteristics of best practices ensured high levels of consensus from the experts and in fact represent the critical factors of the procurement procedures and at the same their distinctive stages. These characteristics highlight the multidimensional and complex process of the procurement of medical devices, indicating that each stage has its own significance and importance. The best result is achieved by a combination of best practices in every stage of the procurement procedures.

The experts achieved high rates of consensus on eight statements with regard to the major problems of the current procurement system. The statements involved *problems, shortcomings and weaknesses identified throughout the various stages* of the procurement process. In fact, the existence of shortcomings highlights the significant room for improvement of the whole procurement chain. Relevant reports and studies also highlight several of these problems. For example, the annual report of the Auditor General of the Republic of Cyprus (Auditor General’s Report, 2010) indicates several cases of expired or inappropriate medical consumables, worth hundreds of thousands of euros, which instantly raise concerns about the lack of inventory management and control systems, the lack of assessment mechanisms and the potential phenomenon of corruption in the supply system. Other findings fall along the lines of the results of other respective research studies (Panayiotou et al., 2004), such as the complexity of medical devices and their high cost necessitating considerable time for the preparation of their specifications and for the reliable assessment of tenders (Terio, 2010). More findings that are interesting include the absence of information systems, which is interrelated with inefficient inventory management and the delayed planning of needs. These findings are repeated in the next research question of the study, which investigates the reasons for the increasing trend of fast-track procurement procedures for medical devices (Table 5). The *lack of coordination/communication between the responsible authorities, the absence of information systems and inventory management and control systems, and the difficulties in timely assessment, planning and prioritization of needs* dominate the experts’ replies in both aforementioned research questions.

The high level of consensus regarding the statement that the suppliers are the main source of “information and influence” on the introduction into the Cypriot market of new products and curative techniques inevitably highlights the influence of the biomedical industry on doctors (Chren, 1999; Brennan et al., 2006; Campbell et al., 2007). The power and influence relationship between doctors and industry seems to be largely present in Cyprus as well. In a relevant question to Cypriot doctors concerning the sources that they take into account when prescribing drugs, 61.1% reported the pharmaceutical sales representatives (Theodorou et al., 2009), who can create conditions that favor influence relationships between doctors and companies. Their reply provides substantial support for the idea that these power and influence relationships flourish in the field of medical devices. This statement, if combined with the absence of a Specialists Committee for the evaluation of requests, allows doctors to develop authoritarian attitudes and adopt practices that could potentially serve their own personal interests simply by invoking their specialized knowledge and experience to justify their requests for specific products. Such attitudes and practices are deeply rooted in this kind of influence relationship between doctors and biomedical technology companies and, usually, lead to increased procurement costs without considerable benefits to the patient. Most importantly, within this conflicting complex of interests, doctors’ professional responsibility is undermined and their relationship with the patients is harmed (Wenger et al., 2000).

Furthermore, there is a particular interest regarding the findings about the strengths of the current procurement system. The experts reached consensus on three issues, namely the *implementation of information systems and the proper organization of the tenders sector, the working experience and diligence of the employees and the legislative framework*. The inability of the experts to reach consensus on more issues perhaps reflects to some degree the significant problems of the procurement procedures, despite the diligent efforts of some employees. Even the positive reference to the current institutional framework has limited value to the extent that it is implemented successfully and any attempts to break the law are punished. Unfortunately, the reports of the Auditor General of the Republic of Cyprus for 2010 and 2011 contain several cases of law violation, mismanagement and failure of suppliers to comply with their contracts’ requirements, without the Government imposing the related penalties, which transform the statements of the experts into an empty shell (Auditor General’s Report, 2010 and 2011). On the other hand, one has to take into account the fact that the content of the legislative framework could provoke unnecessary bureaucratic obstacles and great delays in conducting and completing tenders. This is also highlighted by the experts as one of the main reasons for increasingly resulting to the use of the simplified procurement procedures, which simultaneously poses a contradiction in the views of the experts. In several cases of highly specialized products, the institutional framework could even lead to the establishment of a “closed network” of suppliers with the distinctive features of a monopoly or an oligopoly (Moschuris & Kondylis, 2006).

The launch of the Electronic Procurement Application System for public tenders and the prospect of its complete implementation is considered as one of the first positive and decisive steps. The relevant literature indicates that this initiative has multiple positive effects on the procurement process. It reduces bureaucracy and the processing time for tenders (Panayiotou et al., 2004), it can result to better diffusion of information and facilitate the participation of suppliers in tenders (Ortiz & Clancy, 2003) and, last but not least, it reduces the cost (Erridge et al., 2001; Federici, 2006) and raises the level of transparency and efficiency of the procurement procedures (Andersen Consulting, 2000).

The experts were asked to submit proposals and solutions for improving the procurement system. Seven different statements on the subject matter achieved very high rates of consensus. The implementation of information systems at all the levels of the procurement chain constitutes a self-evident prerequisite for cost containment, product quality, flexibility and efficiency in the procurement mechanism (Ortiz & Clancy, 2003; Ketikidis et al., 2010). A procurement management information system, as an integral part of the integrated hospital information system, provides all the necessary information for planning, organizing and managing hospital medical devices. However, introducing a competent computerized system demands strenuous and detailed grouping of products and a comprehensive and universal coding, something that the experts indicated. Such codification must be neutral and without direct or indirect references to specific products and manufacturers (Ketikidis et al., 2010).

In addition, the cooperation and coordination between hospitals and the relevant departments/units constitute *a sine qua non*, particularly for processes such as procurements/tenders in which a number of people are involved. The aforementioned statement does not only indicate the desired cooperation between services – such as medical, clinical and administrative – but also stresses the need for coordination and optimization at all the stages of the procurement chain (Zhu et al., 2013). As far as the Cypriot case is concerned, the need for cooperation and coordination is evident since there are two different units/departments in the MoH which deal with procurement.

Several proposals from the experts focused on the establishment and operation of committees and mechanisms, either at the hospital or at the ministerial level. These institutions are most promising provided that they are sufficiently backed up and supported and that their functions are not bureaucratic and time-consuming. From a different perspective, the establishment of committees both at the hospital and at the ministerial level could be seen as a waste of resources for a health system with only five public hospitals. The establishment of a well-organized and sufficiently staffed Central Unified Procurement Agency, which would assume the responsibility to conduct central tenders, may lead to large economies of scale. An indicative example of such an agency comes from NHS Scotland, in which more than 200 million euros of savings were achieved in 2006 (NHS Shared Business Services, 2011).

Monitoring for the effective management and evaluation of products is an important element of the procurement chain, although such mechanisms require highly skilled personnel, the use of high technology and a high level of expenditure. For small countries, such as Cyprus, it is cheaper to borrow knowledge and data from other countries with organizations that are exclusively responsible for these matters, such as the National Institute of Clinical Excellence (NICE) of Great Britain, the Federal Institute for Pharmaceuticals and Medical Devices (BfArM) of Germany, the Haute Autorité de Santé (HAS) of France and even the American FDA.

Finally, the implementation of programmes for the continuing education/training of the involved officers for acquiring specialized knowledge and skills in designing and producing/managing complex trading practices in competitions has significant value. Training and information seminars provided especially for the users of such products should not be left solely to suppliers, who often develop influence relationships with them. Training should be the main concern of hospital administrations and the MoH.

The findings of this study are valuable for two reasons. Firstly, they can inform officials about the main problems of the procurement procedures for medical devices in Cyprus; and secondly, they can serve as a guide for their future policy initiatives on the specific subject matter. Further research will be necessary after the implementation of these recommendations for improvement in order to evaluate and compare the factors affecting the planning/management system of medical devices in Cyprus as well as the success of any measures taken.
